# Video recording as a data collection method in vulnerable populations - methodological and ethical considerations

**DOI:** 10.1016/j.pecinn.2025.100432

**Published:** 2025-09-22

**Authors:** Marte-Marie Wallander Karlsen, Kari Sørensen, Berit Hofset Larsen, Lena Günterberg Heyn, Jennifer Gerwing

**Affiliations:** a Department of Postgraduate Studies, Lovisenberg Diaconal University College, Lovisenberggt 15b, 0456 Oslo, Norway; b Centre for Medical Ethics, Institute of Health and Society, University of Oslo, Postboks 1130, Blindern, 0318 Oslo, Norway; c Center for Health and Technology, Faculty of Health and Social Sciences, University of South-Eastern Norway, Grønland 58, 3045 Drammen, Norway; d Health Services Research Unit, Akershus University Hospital, Postboks 1000, 1478 Lørenskog, Norway

**Keywords:** Behavioral research, Children, Ethics, Healthcare, Observation, Patient-clinician communication, Video recording, Vulnerable populations

## Abstract

**Introduction:**

Video recording vulnerable situations in healthcare practice raises ethical challenges that require addressing throughout the research process. Such challenges are linked to protecting research participants and assessing when and how using video recordings is appropriate.

**Aim:**

This article aims to present methodological and ethical considerations inherent in video recording vulnerable participants and to offer future researchers concrete guidance and inspiration as to how they might assess these aspects of their own planned video research.

**Results and discussion:**

As a group of researchers who have used video recordings to collect data of patients in vulnerable situations, we reflect upon our own methodological and ethical choices against the backdrop of other research studies and ethical guidelines.

**Conclusion:**

Video recordings provide a unique opportunity to observe human interaction and to understand more about how we relate to each other. This article contributes to knowledge regarding empirically and ethically sound practice. We have demonstrated how methodological and ethical considerations are intertwined and should be treated as such.

**Innovation:**

There are no existing guidelines or tools specifically for conducting and reporting qualitative studies using video recording that link the ethical considerations to the methodological choices. This article could provide a point of departure for establishing a reflective tool.

## Highlights

• Video recording enables observing human interaction and relationship.
• Addressing ethical challenges is essential when using video recording in research.
• Methodological and ethical considerations in video research are intertwined.
• Methodological and ethical reflection provides guidance and inspiration.
• A standard or checklist that can ensure the ethical quality of video research is required.

## 1 Introduction

Interactions between patients and providers serve as the arena for patient care, during which they share information, make decisions, and establish a relationship. These interactions constitute part of the inner workings of a healthcare system but can be a “black box”, private moments between patients and providers that are impervious to observation. Video recordings of clinical interactions provide a window into the black box by enabling observation of social actions and interactions in an authentic context, offering insight into patient care based on analysis that involves reviewing and scrutinizing the recordings countless times [[1], [2], [3]]. Video recordings capture essential elements in communication, such as gestures, tone of voice and facial expressions. While such richness and detail offer an interpretive advantage, this richness comes at the price of making the participants easily recognizable.

Overarching ethical principles in healthcare research are respect for autonomy, beneficence, non-maleficence, and justice [[4], [5], [6]]. When providers and patients are particularly vulnerable or marginalized, video recording raises specific ethical challenges that require addressing throughout the research process (e.g., protecting research participants, taking responsibility for assessing when and how using video recordings is appropriate) [[7], [8], [9]]. People may be vulnerable for various reasons. In a biomedical context, vulnerable patients might not be able to protect their own interests because of illness, debilitation, immaturity, cognitive impairment, or socioeconomical poverty, or circumstances that decrease the likelihood that others will be alert or sensitive to their interests [4]. Being vulnerable also depends on relational, situational, and personal factors. Taking responsibility and precautions for assessing complex factors and foreshadowing repercussions requires both ethical expertise and reflection [10]. At the same time, the term *vulnerable* should be used with caution, because this term can lead to stereotyping or overprotecting people in certain populations [4]. Indeed, vulnerability can be invoked to exclude groups from research, often explained as a desire to protect these patients [8]. Involving individuals in vulnerable situations, therefore, demands special attention, as these individuals may have “an increased likelihood of being wronged or of incurring additional harm” [6]. After examining the value of using naturally occurring data in vulnerable participants, Drewett and O'Reilly [8] stated that one can empower vulnerable patients to be involved in research while simultaneously safeguarding them.

Although current evidence has not identified significant, detrimental effects on patients while being video recorded, researchers still need to take precautions and implement necessary measures to minimize the possibility that detrimental effects might occur [9]. In this paper, we use our own video observational studies of interactions between providers and patients in vulnerable situations to highlight the precautionary measures we took. These studies included patients who might be considered vulnerable due to physical decline, age, psychological distress, or existential strain. One study included children and their parents interacting with healthcare professionals during a painful procedure. Young children live in the present and cannot always recall or articulate what has happened. However, their reactions and responses from the healthcare providers during the procedure were possible to capture and an important prerequisite for improving practice [11]. The second study included critically ill, mechanically ventilated patients, who struggled to communicate due to sedation and critical illness [12,13]. Learning more about how these patients communicated under these conditions provided detailed descriptions of the interaction and helped build a picture of how to improve clinical practice in similar circumstances. The last study included patients with advanced cancer and their relatives during medical encounters. Patients with advanced cancer may struggle to recollect the details of a conversation during which they had received crucial information. In this study, the patients and companions were recorded while they were in a potentially high state of emotional distress, discomfort, and existential concern [14]. Table 1 provides additional information about the three studies. An extended version of the table is found in Appendix 1.

**Table 1:** Presentation of the studies involving video recordings of vulnerable populations.

Aim	Results
Study 1: Sørensen et al., 2020	
To explore children's expressions of pain and fear during injection training and to explore communication and interaction between children, nurses, and parents.	Children mostly expressed fear indirectly or nonverbally. Anticipatory fear appeared more bothersome than the pain experience itself. Children were observably more relaxed when given time to play with or engage with the equipment. Initiating talk about fear and pain was often a response to children's nonverbal or verbal expressed emotions, when nurses “translated” indirectly stated worries and suggested possible coping strategies. Coping strategies suggested after the child had become distressed (i.e., rather than to more indirect observable fear) were observably less helpful.
Study 2a: Karlsen et al., 2019	
To explore the interaction between mechanically ventilated patients and healthcare personnel in intensive care units (ICUs), with a special emphasis on patients' initiative to communicate.	Successful communication involved two steps: health care provider must (1) notice and attend to the patient's initiation, and (2) understand what the patient wants, which could take extra efforts that exhausted patients. Patients sought the attention *visibly*: waving, gesturing, gaze, facial grimaces. They also sought attention *audibly*: tongue clicking, banging, tapping bedsides, breathing heavily to provoke alarm from ventilator. Patients facilitated understanding using *symbolic gestures* and *pointing gestures* (with hands, eyes, lips, head tilts) to direct attention to objects (e.g., radio, clock). They could also form words with lips. Some used communication aids, but these were rarely successful. Expressed content included psychological, physical, social, and medical treatment domains. Health care providers' responses were immediate or delayed, requiring patients to intensify actions. Understanding was immediate, took time, or lacking.
Study 2b: Karlsen et al., 2020	
To explore how bedside micro-decisions were made between conscious patients on mechanical ventilation in intensive care and their healthcare providers.	There were 142 micro-decisions identified in the material. Six communication patterns of decisions identified (non-invited, substituted, guided, invited, shared, self-determined), which could be placed on a continuum of the degree to which patients were involved. More than half of the micro-decisions were non-invited (the decisions were both initiated and decided by the provider, without explicitly asking for the patient's preference). Three main features in the decision-making processes: how the patients continuously shifted between being in observer or participant positions when interacting, how the patient and the provider negotiated micro-decisions, and how decision-making was limited by the need for energy restoration.
Study 3: Hofset Larsen et al., 2022:	
To explore what existential concerns advanced cancer patients disclose during routine hospital (outpatient) visits, and how they communicate such concerns.	127 utterances fit the definition of conveying existential concerns, which could be categorized into threats to being at the physical, psychological, social, and spiritual levels. Most were patient-initiated. Patients brought up existential concerns indirectly and often with a degree of hesitation, which was observable in features of speech and body gestures (sitting uneasily, frowning, pulling hand over face, gazing away). These signs were notably absent in utterances during which patients were conveying more neutral information. Utterances about biomedical matters were often identifiable as conveying existential concerns. Patients displayed few emotions.

Previous literature reveals a need for guidance and tools supporting ethical reflections in this setting [9]. This article aims to present methodological and ethical considerations inherent in video recording vulnerable participants and to offer future researchers concrete guidance and inspiration as to how they might assess these aspects of their own planned video research.

## 2 Methods

The authors convened several meetings to discuss ethical issues encountered in their research. As qualitative researchers, we also revisited our own reflection notes, field notes, and situations we had experienced as ethically challenging during the research process. During discussions and individual reflection, we examined each phase of the research process through the lens of ethical principles (i.e., respect for autonomy, beneficence, non-maleficence, and justice). We emphasized identifying what might be harmful or advantageous to participants, given that video recording was the data collection method. In the following sections, we present the results of this work against the backdrop of ethical principles, other researchers' experiences, and methodological literature. Table 2 provides an overview of the ethical and methodological choices and shows differences and similarities between the three studies.

**Table 2:** Overview over methodological and ethical decision-making in the three video recording studies.

	**Study 1** (Sørensen et al., 2020)	**Study 2 a, b** (Karlsen et al., 2019, 2020)	**Study 3** (Hofset Larsen et al., 2022)
Aims	To explore children's expressions of pain and fear during injection training and to explore communication and interaction between children, nurses, and parents.	To explore communication and interaction between patients on mechanical ventilation and health care professionals.	To explore what existential concerns advanced cancer patients disclose during routine hospital (outpatient) visits, and how they communicate such concerns.
Recruitment of participants and consent process	Inclusion of 8 children (5–16 years), 11 parents and 7 nurses, in 9 training sessions. A coordinating nurse at the ward informed eligible families about the study and asked whether they wanted more information about the study. Researcher informed children and parents verbally and delivered written information before the procedure. Nurses provided consent to be asked about participation prior to study onset. Nurses, parents, and children (>12 years) provided written consent before the procedure. When one of the parents was not present, consent was given via telephone, and written consent documents were sent afterwards.	Inclusion of 10 patients and 60 health care professionals (nurses, physicians, physiotherapists, radiologists). Department screened eligible patients through a checklist. Researcher then informed patients and relatives, and video recordings were planned for the next day. After the video recordings, patients gave their written consent. Health care professionals were approached either by the management before the recording or asked during the recordings.	Inclusion of 13 patients, five physicians and ten next-of-kin accompanying the patient to the consultation. In a previous project studying patient-physician-communication, 497 medical encounters were videotaped, from which we selected a sub-set. For the original project, participants were recruited widely from various departments. The patients were adults (>18 years and competent to consent) with various cancer diagnoses. In connection with the original project, participants in all videos provided broad consent for use of the videos in further communication studies. This consent was reconfirmed by sending a text message to the participants and the participants responded back.
Data collection	Two Go-pro cameras were placed to capture close-ups of the faces and an overview, plus an additional audio recorder. The procedure was carried out in the child's bedroom on the ward and lasted 8–45 min. Researcher placed the equipment and was present during the procedure. Short interview with participants right after the procedure.	Synchronous video and audio recordings, two sources with different views of the room (surveillance cameras with sufficient quality and FM-band audio recorder) with the patient (one close-up, one full room view) for about 3 h. Researcher mounted the equipment, remained outside of the patient room during the recordings in case of sensitive procedures, acute or adverse incidents. Interviews conducted with providers and patients after the recording (up to 8 months after the ICU stay).	One camera in one angled fixed position. During medical examination, the camera was turned or blinded, so the patient was no longer visible, but it was possible to partially hear the dialogue.
Data management	Followed a data management plan. Uploaded data to an encrypted PC before uploaded to “services for sensitive data” (TSD) for safe storage and data analysis. Signed consent stored in a secure safe.	Followed a data management plan. Data downloaded to “services for sensitive data” (TSD) for safe storage an also analyzed there. Signed consent forms in a secure safe.	The video recordings were stored in a secure server at the hospital where the recordings were conducted, and all observations were carried out at this site.
Data analysis	Transcriptions of talk and behaviour by using F4Transcript. The software program NVivo 11 was used to organize the data and assist the analysis. Inductive analysis using thematic analysis. Interaction Analysis of events, including group session with collaborative viewing and discussion in the research team.	Material transcribed in an excel sheet covering both the vocal and non-vocal actions in the interaction. Selection of segments related to 1) attention seeking actions and then 2) micro decisions. Analyzed through Mangold Interact and narrative descriptions of the interactions.	Dialogue was transcribed into an excel sheet covering vocal and non-vocal actions. Transcripts were encrypted by password, free from personal information that could identify participants, and accessible to the research team only. Both videos and transcripts were used for analyzing the content and the interactional functions of participants' utterances.
Reporting of findings	Illustrating quotations are named as case numbers in the presentation of findings.	Not possible to disguise who agreed to be included in the study based on the data collection method. Fictive names in presentation of findings.	Patients were given pseudonyms, and all physicians are referred to as ‘she’ to protect their identity. Next-of-kin were referred to as the patient's wife/ husband/ mother/daughter in law.
Assessment of needs for additional support for participants	An immediate short interview done. Individual interviews with children and parents after 4–6 months. Some children and parents watched the video during the interview. The children did not like to see themselves scared and anxious. A user collaborator gave valuable comments on the planning and execution of the study.	Highly vulnerable patients with reduced ability to communicate. Patients were invited to watch segments of the videos if they wanted afterwards. Discussions with both patients, relatives and health care professionals about the video recordings, secure management of their right to withdraw.	It was no longer possible to provide additional support because the recordings were conducted earlier. However, when the text message was sent to the participants after the recordings, requesting additional consent, more than 90 % accepted, suggesting that they perceived the burden acceptable.

## 3 Results and discussion

Throughout all stages of the research process, there are opportunities to assess and take actions to secure participants' well-being continually. Ethical principles can help to identify and analyze what is at stake, that is, the value-conflicts. [4]. An example of a value-conflict could be video recording a conversation that relates to the patient's end of life, which could later inform how health care professionals handle existential matters, but which might affect what the patient chooses to talk about. Generating knowledge that might be valuable for future patients might be harmful for the current patient providing the data. Specifying what the principles imply in a particular context can be accomplished by asking the following:

• What are the potential benefits of using video in this project, and for whom?
• What are the potential burdens and risks imposed on participants, and what can researchers do to minimize them?
• What do participants need to make autonomous choices regarding participation?
• Can the use (or avoidance) of video cause injustice, unfair distribution, or representation?

When principles conflict, careful consideration focuses on weighing potential benefits against potential harms of video recording patients and providers. Navigating and making ethically justifiable decisions can be demanding, especially for novice researchers. However, fulfilling ethical demands is not always well operationalized: Research ethics refer to standardized regulations and guidelines researchers must follow, but these sources do not operationalize how the guidelines can be applied in practice [15].

### 3.1 Research aims

When planning to study practice, researchers might begin by reviewing the research aims critically to consider whether video recording is necessary. Does the aim, current knowledge status, and need for further studies justify the use of video recording interactions in the clinical setting, or could other observational methods suffice (e.g., audio recording or taking field notes)? One may find video analysis useful for revealing the specifics of communication practice, and thus in accordance with the principle of beneficence [4]. However, if the potential benefits for future patients are unclear, deciding whether to participate by being video recorded may place an unnecessary burden on the participants, reducing ethical justification due to a breach of the principle of non-maleficence. Even if the aim and research questions justify video recording practice, due to the principle of respect for autonomy, the patients and providers to decide for themselves whether their own participation is potentially meaningful [3,[16], [17], [18]].

### 3.2 Recruiting participants and the process of consent

While Institutional Review Boards (IRB) or Regional Ethical Committees (REC) regulate ongoing research, their approval does not guarantee that participants' well-being is secured, because as a research project unfolds, consent can be relevant in different ways. Despite recruiting participants ethically, word limits constrain authors of published articles from providing details that could guide future researchers. Indeed, Golembieski et al. [3] found that only half of the video recording studies they reviewed included details regarding how the authors of the studies conducted the recruitment.

Respect for autonomy entails a duty to respect the patient's right to self-determination. Autonomy rests on two pillars: voluntariness and agency. According to Beauchamp and Childress [4], autonomous choices are characterized by intention, understanding, and freedom from controlling influences. Understanding requires not just access to sufficient information, but the ability to process that information and weigh the pros and cons of the choice offered. For example, when the study involves video recording, the choice to participate rests on understanding that confidentiality is more difficult to safeguard, as the patient's identity is observable. Controlling influences can be more subtle than the use of force, for example, persuasion, manipulation, withholding information, or providing biased information [4].

#### 3.2.1 Understanding the implications of participating

Providing participants with sufficient time to digest information and consider participation can facilitate autonomous choices, something challenging to accommodate in busy healthcare settings [19]. In the three studies highlighted here, the time to consider varied, from approaching patients the same day as the planned recording to doing so a day earlier. In Hofset Larsen et al.'s [20] study, participating patients with advanced cancer gave provisional consent to be recorded. Later, the participants were contacted via text message, providing time to reconsider full consent post-discharge [21] and removing any sense of obligation to the researchers. In Karlsen et al.'s [12,13] studies involving mechanically ventilated patients, the participants could provide consent initially only by nodding or other non-vocal methods. After discharge from the intensive care unit (ICU), the participants could reconsider their decision and provide their final decision in writing. These two approaches highlight the importance of adapting the consent procedure to the patient's context, well-being, and ability in the moment, all of which are rooted in the ethical principle of autonomy and right to self-determination.

Ethically sound research demands clear communication about what the study entails for potential participants, including potential benefits and harms of using video. However, practical details may also be important, such as the expected recording duration, how video will be collected, and what participating in the recording requires from the participants. As mentioned previously, while research may benefit future patients, health professions, and society at large, that research may not benefit the participants, except for perhaps offering a sense of meaning by contributing to others. Some participants may not realize that participation will not offer better treatment [4]. That said, in their review of studies about vulnerable participants' experiences, Alexander et al. [18] found that most were overwhelmingly positive toward participation. This finding aligns with our experience, as participants frequently expressed gratitude and emphasized how meaningful their involvement was to them. Pino et al. [22] explored views of whether video recording patient–doctor consultations is acceptable for research and training purposes: While some participants reported feeling obligated, perhaps risking freedom of choice, they still expressed that they preferred being asked rather than excluded from recruitment. Their feeling of being seen as competent may override potential vulnerability [9].

When video recording is used for data collection, patients may misunderstand practical issues regarding participation, for example, if the video recording procedure is complex. Karlsen et al. [12,13] discovered that one of the participating patients had misunderstood the time scale of the video recording. He reported later that he imagined the recording happening over three consecutive days, instead of only three hours. When he was able to express himself more thoroughly, he explained: “The second day I waited, wondering where you were… The third day, I understood you would not come.” This misunderstanding was problematic both because he had not fully understood what he had agreed to and because the tracheostomy and critical illness made asking the providers about the procedure extremely difficult.

#### 3.2.2 Consent is a process

Patient autonomy is central in all research designs and is operationalized through consent procedures [4]. Respect for autonomy includes informing participants that they have the right to withdraw from the study at any time without reason or penalty [6]. After initial consent, researchers are responsible for ensuring that consent remains valid as conditions or circumstances evolve. Indeed, the “gold standard” may be to construe consent as a *process* rather than a one-time event [[22], [23], [24], [25]]. Ensuring the participants' right to withdraw requires assessing consent validity continuously in relation to their well-being [9,24]. The consent process also includes time before and after the camera is on, including participants' control of the recordings afterwards. In clinical settings, when video recording is happening, it is necessary to plan how to handle non-participating patients who have not given consent but who could be captured on film [26]. The following example illustrates how ensuring the participant's well-being while not recording other patients became intertwined issues.

For Karlsen et al. [12,13], an ICU patient had agreed to participate, but she had to be transferred to another room the evening before recording to ensure no other patients would be recorded. In practice, moving rooms was time-consuming and exhausting for the patient. The transfer happened only one hour before the planned video recording. When Karlsen mounted the equipment, she observed that the patient was breathing quite rapidly, appearing very anxious and fatigued. She approached the patient, commented on her condition, and suggested that she and the participant cancel the video recording. The patient nodded and agreed, visibly relieved. Ensuring her well-being meant losing a participant. Nevertheless, not burdening the patient was the only ethically sound decision under these circumstances. The ethical principle of preventing harm took priority over her initial consent, as the circumstance s had changed enough to reconsider the validity of the patient's initial consent.

#### 3.2.3 Capacity to consent

In Sørensen et al.'s study of injection training [11], a coordinating nurse recruited the participants, and the researcher approached them on the day of or some hours before the planned procedure. Parents and children received verbal and written information about the study. Before the procedure started, Sørensen returned and received consent from the accompanying parents and children. For most children, only one parent was present. Therefore, Sørensen provided information to the other parent over the phone, asked whether that parent consented, and followed up with written consent documentation afterwards. While no one withdrew from participation and the procedures were considered ethically sound, some cautions are in order. First, whether the parents and children had sufficient time to (re)consider their participation was debatable, as they left the hospital shortly after the injection and patient education. Second, the youngest children may not have really understood what they were agreeing to, and they might have just agreed because their parent did. In such circumstances, assessing capacity to consent is not straightforward. Careful balancing is in order, and sometimes the study's aim and keeping the burden as light as possible for participants will guide researchers' decisions. The recommendation from Pino et al. [22] is to have a risk assessment in place that safeguards participants: excluding participants in apparent distress, avoiding pressure, providing time to consider participation, and offering opportunities to opt out.

### 3.3 Data collection

#### 3.3.1 The quality of visual and audible data

As a patient, being video recorded in vulnerable situations may feel burdensome, even potentially harmful. Ensuring the quality of the data is, therefore, as important ethically as it is methodologically. Good quality includes camera positioning that can achieve good visual access and high-quality audio. Sometimes, measures needed to ensure such quality can conflict with participants' well-being. For example, implementing best quality recordings might be interpreted as too invasive if the cameras and audio recording systems are positioned too close to the interaction, which may require creative solutions. Sørensen et al. [11] used GoPro cameras for video recording because the cameras were small and easy to set up. However, these cameras lacked excellent sound quality, so Sørensen set up an additional, unobtrusive audio recorder. This solution proved very useful, as the children spoke in low voices. Karlsen et al. [12,13] used two FM audio recorders placed in different locations in the room, which ensured good quality audio without disturbing patient care, as the nurses moved freely around the room.

#### 3.3.2 Researcher in the room or not

Our studies illustrate various ways to collect data; each strategy matching the contingencies of the setting. Sørensen et al. [11] discovered that the patients, parents, and healthcare providers moved around in the room during the injection procedure. Therefore, she remained in the room to adjust the camera direction when necessary. Karlsen et al. [12,13] chose a stationary mounting for both the microphones and camera equipment, and she remained outside the room during recording. One camera captured the whole room, and the other focused on the patient and their bed. In Hofset Larsen et al.'s study [14], the researcher was not present during the video recordings and came in only to change the angle of the camera to avoid recording physical examinations. While it may be necessary to intervene by adjusting the camera direction, the researcher's presence might interfere with the natural course of the interaction, and being outside of the room can create space and distance that can be useful in the analytic phase [1].

#### 3.3.3 Participant reactivity

The degree to which the recordings reflect “naturally occurring” interaction depends somewhat on whether the participants' knowledge that they are being recorded influences their actions [22,27]. Several authors describe this phenomenon as “participant reactivity”: participants may alter their behaviours, which has implications for what is being analyzed and what the results represent [[28], [29], [30]]. In Karlsen et al.'s recordings [12,13], one of the participants clearly addressed the camera by looking into it and shaking his head in frustration when the nurse did not observe his attempts to communicate. In healthcare settings, how the presence of a camera influences the interaction is also an ethical question, since participants may adjust conversational topics in response. For example, in one of the recordings from Hofset Larsen et al.'s study [14], the physician left the room briefly, and the patient and his wife began a private, quiet conversation. Suddenly, the wife looked toward the camera and made a hush signal to her husband, closing the conversation. She demonstrated that video-recording this moment changed what she and her husband felt comfortable discussing, potentially affecting his care.

#### 3.3.4 Decreasing sensitivity

Participant reactivity may not be linked solely to the use of recording equipment, as observation awareness might also occur using traditional observational methods [26]. In any case, making the participants comfortable being observed may decrease their anxiety, thereby reducing reactivity as well [26,31]. Haidet et al. [27] performed a “sensitivity session” where they collected five videos for each patient and never used the first. Karlsen et al. [12,13] had just one session for each patient, but the sessions typically lasted over three hours, giving both the patients and healthcare professionals a chance to become less focused on the technical equipment. Sørensen et al. [11] found that talking about the camera functioned as an icebreaker for the participants, appearing to make them feel more comfortable. Such small incidents also provided a break with an element of humor during a needle procedure that often was frightening for the children. Sørensen et al. [11] also had shorter segments to record, with one specific procedure of interest to investigate. Her experience was that the participants were more focused on the complex procedure she was recording than on the camera or her. When she asked the participants about their experience of being recorded, children, parents, and nurses claimed that they quickly forgot about the cameras. While Antal et al. [31] observed slight evidence of participant awareness during video recordings in their study, it did not seem to influence the communication in the clinical visit. For the healthcare personnel, camera awareness also decreased significantly as they participated in more recordings.

### 3.4 Data analysis and reporting findings

Choosing methods for analyzing video recordings depends on the research questions, and such choices are often a complex decision, no matter which methodological approach you choose.

#### 3.4.1 Initial ethical considerations during the analysis process

The analysis of video recordings is unique in that it requires a degree of transformation of what patients and physicians said and did. The researcher's ethical responsibility is to be faithful, systematic, and fair in those interpretations and subsequent decisions and categorizations. Analytical methods with a stronger ethical footing are those that have theoretical consistency in how speech, interactional cues, and communication behaviour are interpreted and that are traceable, carefully documented, and transparent [1,32]. While many well-established coding systems already exist [33], if no previously developed coding system can address the research aims, one can analyze inductively, drawing on conversation analysis [[34], [35], [36]] or other interaction analysis traditions [32,37]. Often, analytical approaches can be combined. Sørensen et al. [11] explored expressions of fear and pain as well as the interaction and communication between child, parents and nurse. The authors used an inductive thematic analysis [38] combined with interaction analysis [37]. However, when interpreting children's cues of negative emotions (fear and pain), the authors used the Verona coding definitions of emotional sequences (VR-CoDES) to justify their own interpretations [39]. While Hofset Larsen et al. [14] used microanalysis [32] for an inductive analysis of how patients disclosed existential concerns, these authors used the simplified version of the Jeffersonian system for transcription to capture details beyond the participants' words, as the video would have to be deleted before the analysis was completed. Beyond considerations such as these, one can choose from a variety of qualitative and quantitative methods that can be used inductively or deductively [1,37]. According to Jordan and Henderson [37], one way to ensure a thorough coding and analysis process is to arrange a collective viewing for the entire research team, where they can explore and reflect on the events together and agree on a common interpretation. Such procedures can be especially important in video analysis, where small behavioural or vocal details can change the interpretation of the events [32,[34], [35], [36], [37]]. From our experiences, collaboration has been an invaluable part of the analytic process, especially when performing inductive coding not derived from a standardized and previously tested coding scheme.

#### 3.4.2 Stimulated recall interviews

Exploring participants' recollections and explanations regarding the recorded interactions may be of interest. The video recordings can be shown to the participants to stimulate memories and create an additional source of data [17]. This type of analysis changes the participants' experience of the interactions and of themselves. Conducting stimulated recall interviews with vulnerable patients should only be undertaken if potential results of such an approach directly support the study's overall aim, offers unique insights that cannot be obtained through other methods, and if the procedure for doing stimulated recall ensures the participants' well-being throughout the interview process. Karlsen et al. [12,13] conducted, in addition to the video recordings, interviews with the patients after the patients were discharged from the ICU to gain a deeper understanding of their experiences. As many patients might struggle to remember their ICU stay due to their illness, the participants were offered the opportunity to view some segments of their recording. The patients then experienced and reflected upon their own illness differently than what they had remembered, as they observed their own appearance, the room from a different angle, and their intensive care treatment. In some moments of video recording, they observed their interactions with the health care professionals, illustrating the patients' struggles to communicate their needs, which verified their experiences. In the immediacy of a stimulated recall interview, such situations require sensitive and constructive handling. Sørensen et al. [11] experienced likewise when showing segments from the video to children and parents during an interview four to six months after the injection training. Rather than showing segments to elicit participants' interpretations, Sørensen et al.'s purpose was simply to offer a viewing opportunity. For example, since most children had only one parent present during the procedure, the parent who did not attend might be curious to watch what happened. The parents expressed a positive experience of seeing the child in the previous procedure, while the children visibly did not like seeing themselves in such a vulnerable situation. They had developed increased coping and security with the injections and expressed embarrassment when they viewed themselves in these early experiences. Although stimulated recall using video recordings could benefit patients by offering a deeper understanding and meaning, it could be harmful, as it might make the patients recall traumatic experiences. Our experiences also illustrate that participants differ in their interests, reasons, and experiences of participating or wanting to see the recordings, which neither the researcher nor participant may be aware of in advance. We would therefore advise caution when offering participants this opportunity, with critical reflection weighing the possible benefits and harms.

#### 3.4.3 Confidentiality and the vulnerability of clinical practice

Results of a study can be maximized when excerpts from the interactions can serve as illustrations of actual practice for teaching or other modes of dissemination. Aligned with previous literature [9,26,31], all our studies demonstrated how health care professionals themselves become vulnerable when they agree to have their practice video recorded and scrutinized. Their professional behaviour is observed through a research lens, exposing how they manage very complex situations. To safeguard professionals' anonymity, one can use transcripts and avoid descriptions of their personal details and communication style. Nevertheless, breaching confidentiality is one of the significant risks in video-based research because collected data reveals individual, identifiable characteristics. Not ensuring anonymity may harm participants as well as future research due to loss of trust.

Certain recorded situations may illuminate less favorable sides of clinical practice, such as performing a procedure with an obviously reluctant patient or an agitated child. One can frame these situations negatively (e.g., as failing to ensure patient autonomy) or as highlighting the variety of ways professionals handle everyday practice challenges. Avoiding judgment of the professional's behaviour can be important for preventing unnecessary harm or for not holding them to unrealistic standards, given the many unforeseen happenings in clinical practice. Indeed, some participants commented to the authors about being recorded in difficult situations or being unsatisfied with their performance. How the researcher handles difficult moments of practice and related confidentiality issues will affect the analytical process, the relationship with participants, and the obligation to protect their anonymity. For examples to be presented in articles, illustrating moments of best actual practice puts the participants in a better light and may better serve educational purposes. In addition, if descriptions of interactions are rooted in what is observable, one can avoid value-laden words (both negative and positive).

### 3.5 Assessment of needs for additional support for participants

As mentioned previously, interaction research is primarily beneficial for future patients, healthcare providers, educators, and society- not the participants themselves. The fact that the research does not benefit participants themselves directly amplifies this imbalance. Hence, the threshold for what we should accept as participants' burden or harm should be higher than what we would accept if the research would benefit the participant [6]. Anticipating what additional support the participants might need when they agree to being video recorded can be difficult. Involving children or adolescents may also introduce challenges to anonymity, as social media has become a natural part of people's everyday lives. Tutenel et al. [25] reported that some of their teenage participants wanted their names disclosed because they might want recognition or the opportunity to make the video public. Unexpected requests for ownership of the data may also occur, perhaps because the patients feel connected to the video-recordings that tell their story. Patients might ask for a copy of the video recordings, which may contain extremely sensitive data not only about them, but also about the professionals or others involved in the videos. Such a situation occurred in Karlsen et al.'s study [12,13].

### 3.6 Innovation

Using our insider knowledge of how we conducted our studies in different settings, we illustrate the complexity of ethical and methodological choices when using video recordings between vulnerable patients and healthcare providers. While researchers have a responsibility to assess possible harms and benefits for participants continuously and to respect their right to autonomous choices, we aim to inspire future researchers to use video recordings to provide valuable knowledge within clinical settings, including vulnerable participants. Collective research expertise evolves every time a research team makes its systematic reflections transparent, which allows others to learn from their experiences, whether positive or negative. Future generations of researchers and review boards would benefit from a reflective tool that would support excellence in video-based research while integrating the intertwined methodological and ethical issues.

### 3.7 Conclusion

Video recordings provide a unique opportunity to observe human interaction and to understand more of how we relate to each other in complex health care situations. Based on our experiences, which aligned with the literature, we believe it is important to consider seriously the use of video recordings when investigating certain topics, as neglecting this method could potentially overlook valuable insights. Simultaneously, video-based research raises unique ethical challenges for researchers to address to secure participants' well-being. We conclude that one cannot separate methodological considerations and choices from the ethical considerations, context, or the patient population. That is, the most robust study design incorporates state-of-the-art methodological and ethical qualities. Some key recommendations from the authors are as follows:

• Review the research aims critically to consider whether video recording is necessary.
• Invest time in planning the details of the consent process, how to respond to evolving situations that have an impact on consent, and how to secure the participants' well-being during data collection.
• When presenting the study to potential participants, highlight both the benefits for future patients and the potential harms associated with using video recordings.
• Identify potential threats to confidentiality and anonymization of the participants and how these threats can be minimized.
• Identify the limitations and possibilities of patient participation in the study context.
• Develop the methodological choices with a clear reference to ethical principles on which the study is founded.
• Report the ethical considerations during the study in a transparent way, as these deserve special attention whilst including vulnerable populations.

## CRediT authorship contribution statement

**Marte-Marie Wallander Karlsen:** Writing – review & editing, Writing – original draft, Project administration, Methodology, Investigation, Formal analysis, Data curation. **Kari Sørensen:** Writing – review & editing, Writing – original draft, Methodology, Formal analysis, Data curation, Conceptualization. **Berit Hofset Larsen:** Writing – review & editing, Writing – original draft, Methodology, Investigation, Formal analysis, Data curation, Conceptualization. **Lena Günterberg Heyn:** Writing – review & editing, Writing – original draft, Methodology, Formal analysis, Data curation. **Jennifer Gerwing:** Writing – review & editing, Writing – original draft, Methodology, Formal analysis, Data curation, Conceptualization.

## Funding

None

## Declaration of competing interest

The authors declare that they have no known competing financial interests or personal relationships that could have appeared to influence the work reported in this paper.

## Appendix A Supplementary data

Supplementary material Image 1
