# Supplementary material for: Video recording as a data collection method in vulnerable populations - methodological and ethical considerations
Source: PEC Innov. 2025 Sep 22;7:100432. doi: 10.1016/j.pecinn.2025.100432 (PMC12538683; doi:10.1016/j.pecinn.2025.100432)
Supplement: Supplementary file 1 — Supplementary material [file mmc1.docx]

**Appendix 1:** Value of video recording as illustrated in the three studies of actual practice

|  | **Why the study is worthwhile** | **Why video recordings are required** | **Results** |
| --- | --- | --- | --- |
| Study 1: Sørensen et al., 2020 | | | |
|  | Managing children’s pain and suffering around receiving injections is best informed by recordings of actual practice.  The way nurses relate to children and parents during training sessions and how they manage fears and worries may affect how future injections are  managed at home. | - to identify and characterize visible aspects of children’s anticipatory fear, reaction during the injection, response afterwards. - to capture the enactment of the procedure (part of the educational process before injection). - to capture the injection itself. - audible aspects of the situation difficult to impossible to interpret without observing precise sequential relationship with children’s, parents’, and nurses’ nonverbal behaviours - to ensure methodological rigour: Also, video record key for *identifying* social processes, *describing* and *making sense of* participants’ conversations and behaviour, *involving* participants and co-authors in observation and analysis, *ensuring transparency* with of results | Children mostly expressed fear indirectly or nonverbally.  Anticipatory fear appeared more bothersome than the  pain experience itself.  Children were observably  more relaxed when given time to play with or engage with the equipment.  Initiating talk about fear and pain was often a response to children’s nonverbal or verbal expressed emotions, when nurses “translated” indirectly stated worries and suggested possible coping strategies.  Coping strategies suggested after the child had become distressed (i.e., rather than to more indirect observable fear) were observably less helpful. |
| Study 2a: Karlsen et al., 2019 | | | |
|  | Patients on mechanical ventilation experience “voicelessness” that can be emotionally distressing.  For ICU patients, meaningful encounters may inspire hope, resources, and motivation to endure a critical time when their lives are at stake.  Knowing how patients try to achieve attention and what they communicate about are important for understanding their care stay as it occurs. | - to identify visible attention seeking actions, which were primarily visible - to recognize and identify audible attention seeking actions, their status required the context video provided - to interpret patients’ meaning, which required observing patients’ attempts to convey it and their responses to health care providers attempts to verbalize it (confirming or not). - to capture where health care providers were and what they were doing (e.g., flushing central venous line, adjusting settings on ventilator) - to capture use of communication aids (e.g., alphabet board, pen and paper) - to capture patients’ bodily signs after unsuccessful attempts at getting attention or conveying meaning (e.g., resignation, frustration, irritation or exhaustion) - to provide an opportunity for other researchers (co-authors) to watch for themselves and provide their own interpretations and for the research group to discuss. | Successful communication involved two steps: health care provider must (1) notice and attend to the patient’s initiation, and (2) understand what the patient wants, which could take extra efforts that exhausted patients.  Patients sought the attention *visibly*: waving, gesturing, gaze, facial grimaces. They also sought attention *audibly*: tongue clicking, banging, tapping bedsides, breathing heavily to provoke alarm from ventilator.  Patients facilitated understanding using *symbolic gestures* and *pointing gestures* (with hands, eyes, lips, head tilts) to direct attention to objects (e.g., radio, clock). They could also form words with lips. Some used communication aids, but these were rarely successful.  Expressed content included psychological, physical, social, and medical treatment domains.  Health care providers’ responses were immediate or delayed, requiring patients to intensify actions. Understanding was immediate, took time, or lacking.  For patients to demonstrate they were giving up after repeated, unsuccessful attempts at conveying meaning they had to turn head away, avoid eye contact, dismiss with a hand wave. |
| Study 2b: Karlsen et al., 2020 | | | |
|  | In a very critical phase of their lives, mechanically ventilated patients experience reduced ability to participant in decisions about their treatment and care, including micro-decisions.  Micro-decisions in ICUs include mechanical ventilation (weaning attempts from ventilatory support, use of tracheostomy speaking valve), symptom management, mobilization, or other procedures (i.e. wound  care). | - to identify micro-decisions health care providers made that involved visible actions with little to no verbal accompaniment - to analyze patients’ involvement in micro-decisions during their care in the ICU, which could only be ascertained through what was visible. - to be able to identify when patient’s initiated decisions, which required observing and analyzing their visible actions. - to be comprehensive and capture the range of micro-decisions, which was necessary to gain sufficient insight to synthesize the results into themes - to provide an opportunity for other researchers (co-authors) to watch for themselves and provide their own interpretations and for the research group to discuss. | There were 142 micro-decisions identified in the material  Six communication patterns of decisions identified (non-invited, substituted, guided, invited, shared, self-determined), which could be placed on a continuum of the degree to which patients were involved.  More than half of the micro-decisions were non-invited (the decisions were both initiated and decided by the provider, without explicitly asking for the patient’s preference).  Approximately one quarter were invited (the patients were asked to express their opinion about the decision at stake).  Three main features in the decision-making processes: how the patients continuously shifted between being in observer or participant positions when interacting, how the patient and the provider negotiated micro-decisions, and how decision-making was limited  by the need for energy restoration. |
| Study 3: Hofset Larsen et al., 2022: | | | |
|  | Advanced cancer poses a threat to all aspects of being, including existential matters  Patients disclosures of existential concerns is rarely investigated, including what they disclose and how. | - to capture any coordination and mutual influence mediated by visible aspects of communication - to be able to note visible expressions and bodily conduct throughout the interaction that could shed light on the meaning and import of utterances identified as disclosing existential matters - to capture visible aspects that the participants would have had access to (i.e., what each made publicly available), for example, expressions of emotion, gaze direction (whom the speaker was directing talk to, where the addressee was looking). | 127 utterances fit the definition of conveying existential concerns, which could be categorized into threats to being at the physical, psychological, social, and spiritual levels. Most were patient-initiated.  Patients brought up existential concerns indirectly and often with a degree of hesitation, which was observable in features of speech and body gestures (sitting uneasily, frowning, pulling hand over face, gazing away). These signs were notably absent in utterances during which patients were conveying more neutral information. Utterances about biomedical matters were often identifiable as conveying existential concerns.  Patients displayed few emotions. |
